# Exploring the perceptions of nursing internship students and their faculty mentors regarding the complexities and hindrances associated with implementing the nursing process within the clinical setting: An in-depth qualitative investigation

**DOI:** 10.1016/j.heliyon.2024.e31715

**Published:** 2024-05-29

**Authors:** Amir Shahzeydi, Zahra Hadian Jazi, Sima Babaei

**Affiliations:** a Pediatric Cardiovascular Research Center, Cardiovascular Research Institute, Isfahan University of Medical Sciences, Isfahan, Iran; b Nursing and Midwifery Care Research Center, Isfahan University of Medical Sciences, Isfahan, Iran

**Keywords:** Nursing process, Obstacles, Challenges, Nursing students, Qualitative research

## Abstract

**Background:**

Utilizing the nursing process within clinical settings serves to enhance the scientific stature of the nursing field. Nonetheless, various research findings suggest that nursing internship students encounter difficulties when it comes to implementing the nursing process and may lack the necessary proficiency.

**Objectives:**

The aim of this investigation is to identify the perceptions of nursing internship students and their faculty mentors regarding the complexities and hindrances associated with implementing the nursing process within the clinical setting.

**Design:**

A qualitative content analysis**.**

**Participants:**

Nursing internship students and their faculty mentors from the Isfahan School of Nursing and Midwifery.

**Methods:**

In this study, individual interviews were conducted with 13 participants. The data for this study were gathered through these semi-structured interviews and subsequently analyzed using the Granheim and Lundman method. To ensure the validity and reliability of the data, the evaluative criteria of Lincoln and Guba were employed.

**Results:**

The obstacles unveiled in this study can be categorized into three primary domains, each with its own subcategories: 1-Student role ambiguity (1-1 Lack of practical autonomy; 1–2 Insufficient proficiency in the nursing process; 1–3 Motivational deficiency). 2- Organizational Challenges (2-1 Shortage of nursing staff; 2-2 Suboptimal interpersonal dynamics); And 3- Gradual Erosion of the Nursing Process.

**Conclusion:**

Recognizing the paramount importance of the nursing process in enhancing patient care quality is universally accepted. Therefore, it is imperative to systematically identify and tackle the challenges associated with its application. This study highlights that these challenges stem from various factors, including the multifaceted roles assumed by nursing students, organizational shortcomings within healthcare institutions, and the neglect or erosion of the nursing process in specific clinical settings. Addressing these issues is crucial for ensuring the effective utilization of the nursing process within the nursing profession and for optimizing patient care outcomes.

## 1 Introduction

The nursing process has been important in the field of nursing for a long time [1]. Hall (1955), Johnson (1959), Orlando (1961), and Weidenbach (1963) were among the first to use the nursing process to describe nursing practice [2]. Since then, theorists have attempted to align their theories with clinical practice. Since 1960, the nursing process has dominated nursing education [1]. Nursing process has gained widespread acceptance as a scientifically grounded method for enhancing the quality of nursing care. This indicates that nurses can only implement evidence-based care in the clinical departments of the hospital through the nursing process [3]. The nursing process is a systematic method that enables nurses to deliver effective care by using scientific reasoning, problem-solving skills, and critical thinking [4]. In simpler terms, it can be seen as a blend of both the artistic and scientific aspects of nursing. Because in the nursing process, interventions should be based on valid references, and prioritizing interventions based on patient needs demonstrates the nurse's expertise and art. Implementing the nursing process at a national level yields economic advantages by increasing patient contact time, lowering healthcare expenses, and reducing hospital stays [5]. In this regard, Bening et al. (2016) referenced an intervention study in which the implementation of the nursing process resulted in a reduction in the length of patients' hospital stays from 6.44 to 5.37 days, an improvement in the average quality of discharge education from 2.24 to 12.2, and an increase in the average quality of document filing by nurses from 2 to 4.23 [6].

Despite its advantages, the nursing process implementation receives insufficient attention [7]. According to Baraki et al.'s study, most nurses did not put the nursing process into practice [8]; In another study, it was found that 71 % of nurses possessed substantial theoretical knowledge about the nursing process, yet a significant portion (42.9 %) did not apply it in practice [9]. Indeed, research indicates that both practicing nurses and nursing students often overlook the importance of the nursing process [10]. And in this regard, several studies have shown that students encounter difficulties in utilizing the nursing process [11,12].

In Iran, the nursing process is introduced during the 3rd semester of nursing education. However, research suggests that its systematic application is quite limited within the country [13]. Indeed, nursing students, both during their internship courses and after graduation, tend not to apply the nursing process for care standards due to a range of challenges they encounter in this regard [14]. Several challenges contribute to the limited utilization of the nursing process by nursing students and graduates, these challenges include: lack of familiarity with the nursing process stages [15], dependency on other nurses and a lack of critical thinking [16], insufficient knowledge [17], inadequate supervision [18], limited access to clinical facilities and equipment, inconsistent and irregular curriculum for teaching, inadequate student evaluation methods [19], insufficient educational opportunities [20], and inadequate clinical training [8].

It's important to highlight that these challenges are particularly pronounced for final year nursing students during their nursing internships. This transition period from student to novice professional nurse is marked by inexperience and high levels of stress [13,21]. The study suggests that qualitative investigations conducted by internship students could be beneficial in uncovering the challenges associated with implementing the nursing process [22]. Failure to identify and address challenges related to the application of the nursing process in clinical settings can have significant consequences. These may include diminished job satisfaction, a decrease in the perceived value of the nursing profession among both nurses and nursing students, an excessive reliance on doctors, unquestioning obedience, adherence to routine actions without critical thought, and ultimately, a decline in the overall quality of nursing care [23].

Given the significance of the nursing process and the negative consequences associated with its inadequate implementation, especially among nursing internship students, research studies strongly recommend conducting investigations aimed at identifying the challenges and obstacles hindering its correct application by these students [22,24]. In contrast to quantitative research, qualitative research offers researchers greater opportunities to uncover and elucidate the intricacies of the clinical environment. It enables a deeper understanding of challenging facets related to nursing process instruction and grants insights into the real dynamics within the field. Qualitative research provides a valuable model for modifying and enhancing existing processes, contributing to practical improvements [25]. Hence, the primary objective of the present study was to conduct a qualitative investigation aimed at uncovering the firsthand experiences of internship students and their supervising professors, shedding light on the challenges and obstacles encountered in implementing the nursing process within the clinical environment.

## 2 Methods

### 2.1 Study design

This research employed the qualitative content analysis method with a conventional approach. Qualitative content analysis has emerged as a widely utilized and methodologically rigorous approach for systematically analyzing textual data in qualitative research [26]. It offers a flexible framework for exploring and interpreting the meanings embedded within the data, making it particularly suitable for investigating complex phenomena such as the challenges associated with the nursing process. In the context of nursing research, qualitative content analysis provides a means to capture the richness and depth of experiences, perspectives, and practices of healthcare professionals involved in the nursing process [27]. By systematically coding and analyzing textual data from various sources such as interviews, focus groups, or written documents, researchers can uncover patterns, themes, and insights that inform our understanding of the complexities inherent in nursing practice.

In summary, qualitative content analysis was selected as the methodological approach in our study due to its systematic and rigorous framework for analyzing textual data, its flexibility in capturing diverse perspectives, and its ability to uncover nuanced insights into the challenges associated with the nursing process. By employing this method, we aimed to contribute meaningful findings to the literature and advance our understanding of nursing practice in contemporary healthcare settings.

The study was conducted in educational hospitals affiliated with Isfahan University of Medical Sciences (Amin, Kashani, and Al-Zahra hospitals), as well as Isfahan School of Nursing and Midwifery. The study's participants consisted of nursing internship students (7th and 8th semesters) and their supervising professors. In Isfahan Nursing and Midwifery school, experienced professors, typically holding the rank of instructor or assistant professor, supervise and evaluate internship students. They monitor each student a minimum of three times in 20 shifts and assess them using predetermined and validated nursing process forms. Interviews were conducted with professors who voluntarily participated in the study.

### 2.2 Inclusion criteria

For students, inclusion criteria were as follows:

1. Nursing students enrolled in the internship course (7th and 8th semesters).
2. At least one month elapsed since the initiation of their internship course.
3. Participants were required to provide informed consent and express a willingness to participate in the study.
4. All participants were students of Isfahan Nursing and Midwifery Faculty, undergoing their internships in teaching hospitals affiliated with this university.
5. Participants were required to have successfully completed the course on the nursing process, which is taught theoretically during the second semester for students.

For instructor and supervisor professors, the inclusion criteria were:

1. Faculty members of the nursing and midwifery department at Isfahan University.
2. A minimum of one semester of experience supervising internship students.
3. Consent and willingness to participate in the study were required.
4. The individual's rank should be either instructor, assistant professor, or associate professor.
Participants who withdrew from the study for any reason were excluded.

### 2.3 Sampling

Participants were purposefully selected among students and supervising professors. This method, suitable for qualitative research, aims to maximize data depth and richness [28] In fact, Recent literature emphasizes that qualitative research, particularly in its interpretive nature, often involves purposeful sampling to target specific experiences or perspectives relevant to the research question [29,30]. Purposeful sampling allows researchers to delve deeply into nuanced phenomena, prioritizing depth over breadth in understanding.

### 2.4 Calculation of sample size

In qualitative research, achieving data saturation is fundamental for ensuring the comprehensiveness and reliability of study findings. Data saturation entails the repetition and validation of previously gathered data, with a focus on identifying recurring themes and confirming acquired information rather than sampling based on statistical parameters [31]. In our research, data saturation was attained following interviews with 10 students and 3 professors, guided by the principle that no new information or themes were emerging from the data [32]. This criterion is crucial in qualitative research, indicating that a comprehensive understanding of the phenomenon under study has been reached [33]. To ensure rigor and trustworthiness in determining data saturation, we employed various strategies such as ongoing data analysis, peer debriefing, and member checking [34]. These iterative processes allowed for systematic review and analysis of interview transcripts until theoretical saturation was achieved.

Overall, our selection of participants and the attainment of data saturation were guided by the principles of purposeful sampling and theoretical depth, aiming to capture diverse perspectives and enhance understanding of the challenges associated with the nursing process. Through rigorous qualitative research methods, we upheld the credibility and trustworthiness of our findings.

### 2.5 Data collection method

The primary method of data collection in this study involved individual semi-structured interviews. During these interviews, participants were invited to share their experiences regarding the challenges and hindrances associated with implementing the nursing process in hospital settings. The initial questions posed were broad and open-ended, with subsequent, more detailed questions emerging as the conversation progressed. Sample interview questions included:

1. Can you describe your familiarity with the nursing process? Or Describe your initial encounter with the nursing process. When did you first become familiar with it? Please elaborate.
2. What are your thoughts on using the nursing process?
3. What advantages do you see in utilizing the nursing process?
4. Can you discuss specific obstacles or difficulties you have faced when utilizing each stage of the nursing process (assessment, diagnosis, planning, implementation, evaluation) in clinical practice?

Follow-up questions were also posed to seek clarification based on the participants' responses. Additionally, professors were invited to discuss their experiences in evaluating internship students and any issues they had encountered regarding the nursing process.

Following each interview, the gathered data were analyzed, and the information was compared with previously collected data. The duration of the interviews varied, lasting between 30 and 45 min, depending on the participants' conditions and willingness. The interviews were scheduled at times when the students were not on their internship shifts, and the interview locations were chosen based on mutual agreement, considering factors such as physical conditions, temperature, ventilation, lighting, and compliance with health protocols during the COVID-19 pandemic.

### 2.6 Data analysis

The data underwent analysis using conventional content analysis, following the five-step process outlined by Graneheim and Lundman [35]. The data analysis in this study followed a comprehensive five-step process, guided by Graneheim and Lundman's approach:

1 Transcribing: Initially, the interview text was meticulously transcribed verbatim from the recorded audio.
2 Meaning Unit Identification: The audio recordings were replayed multiple times, while the handwritten texts were thoroughly reviewed. During this step, the text was segmented into meaningful units, identifying significant portions.
3 Code Extraction: Abstracting semantic units and selecting codes were the next steps. Overt and implicit concepts were pinpointed from the participants' statements, usually as sentences or paragraphs, which were then denoted as codes. Coding and summarization were performed at this stage.
4 Categorization: Codes indicating a common theme or subject were grouped together based on continuous comparison for similarities, differences, and relevance. This process led to the creation of subclasses and main classes, with core codes emerging.

Interpretation and Conceptualization: At the interpretation level, summary classes were identified, and the central concept of each class was determined. Main and abstract concepts were extracted. MaxQDA software was employed in our data analysis process to facilitate systematic coding and organization of qualitative data, ensuring methodological rigor and enhancing the transparency of our analysis [36]. Specifically, the software was utilized to organize interview transcripts, code segments of text based on themes and patterns, and facilitate the identification of recurring themes and emerging insights [37].

Regarding validation and reliability, several strategies were employed to ensure the credibility and trustworthiness of our findings. These included members checking, peer debriefing, and prolonged engagement with the data to confirm the consistency and accuracy of our interpretations [38]. Additionally, the use of multiple coders and the establishment of inter-rater reliability further strengthened the reliability of our analysis [39]. In summary, the utilization of MaxQDA software enhanced transparency in our data analysis process, while validation and reliability were ensured through various methodological strategies. These efforts aimed to uphold the quality and credibility of our qualitative findings.

### 2.7 Data accuracy and strength

In our study, Lincoln and Guba's criteria of trustworthiness, including credibility, transferability, dependability, and confirmability, were rigorously addressed to enhance the validity and reliability of our qualitative findings [40].

1. Credibility: To ensure credibility, various strategies were employed, such as member checking, prolonged engagement with the data, and triangulation of data sources [41]. Member checking involved returning to participants with summarized findings to validate interpretations and ensure accuracy [38]. Prolonged engagement with the data allowed for a deep understanding of participants' perspectives, while triangulation ensured that findings were corroborated across multiple sources, such as interviews and field observations.
2. Transferability: Transferability refers to the extent to which findings can be applied to other contexts or settings [40]. In our study, thick description of the research context, participants, and data collection methods was provided to enhance transferability [38]. Additionally, detailed documentation of the research process allowed readers to assess the applicability of findings to their own contexts.
3. Dependability: Dependability relates to the stability and consistency of findings over time and across researchers [40]. In our study, dependability was ensured through careful documentation of research procedures, including data collection and analysis techniques [38]. Moreover, multiple researchers were involved in data analysis, and inter-rater reliability was established to enhance the dependability of interpretations [39].
4. Confirmability: Confirmability refers to the objectivity and neutrality of findings, ensuring that they are not influenced by the researchers' biases or perspectives [40]. In our study, reflexivity was practiced throughout the research process, with researchers reflecting on their own biases and preconceptions [38]. Additionally, an audit trail was maintained to document decision-making processes and enhance transparency, further ensuring confirmability [41].

## 3 Results

Ten undergraduate nursing students and three supervising professors were involved in the research. Table 1 displays the demographic details of the participants.

**Table 1:** Demographic information of the participants.

Participants	participant	Age	Sex	Grade
P1	Student	22	Female	Bachelor
P2	Student	23	Female	Bachelor
P3	Student	23	Male	Bachelor
P4	Student	22	Female	Bachelor
P5	Student	23	Female	Bachelor
P6	Student	23	Female	Bachelor
P7	Student	22	Male	Bachelor
P8	Student	23	Male	Bachelor
P9	Student	22	Female	Bachelor
P10	Student	21	Male	Bachelor
P11	Faculty	28	Male	Master
P12	Faculty	38	Female	PHD
P13	Faculty	46	Female	PHD

The study's findings revealed three overarching themes, along with their respective subcategories, pertaining to the challenges and obstacles encountered when implementing the nursing process in clinical settings, as perceived by nursing students and professors (see Table 2).

**Table 2:** Subclasses and themes obtained from interviews with participants.

Theme	Sub Classes
1.Student role ambiguity	1.1 Lack of practical autonomy
	1.2 Insufficient proficiency in the nursing process
	1.3. Motivational deficiency
2.Organizational Challenges	2.1 Shortage of nursing staff
	2.2 Suboptimal interpersonal dynamics
3.Gradual Erosion of the Nursing Process	

### 3.1 Student role ambiguity

One of the primary factors identified during interviews with nursing students regarding the non-implementation of the nursing process is the dynamic nature of their role.

Nursing students in their 7th and 8th semesters bear multiple responsibilities. At times, they are expected to fulfill tasks as students, necessitating ongoing learning and training. Concurrently, they are also perceived as recent nursing graduates and novice professionals, with corresponding expectations of competent performance. However, patients often exhibit hesitancy when interacting with these students. Due to their student status, patients may withhold cooperation in care procedures or withhold consent for invasive interventions to be performed by students. These multifaceted roles create challenges and obstacles for students in applying the nursing process.

This category encompasses three sub-categories, which are detailed below.

#### 3.1.1 Lack of practical autonomy

One factor hindering nursing students' effective use of the nursing process is their limited practical autonomy. Many view them as having restricted authority, leading to challenges in care implementation, interprofessional coordination, and follow-up care. Nursing students often require collaboration from colleagues, patients, or doctors for various care interventions. However, due to their student status, they may not be taken seriously, leading to a lack of influence in patient care plans and a need to comply with managerial directives. This lack of autonomy can impede their ability to advocate for their rights, as expressed by a participant.

*“Patients place greater trust in nursing personnel and cooperate fully with them, but they perceive us (students) as inexperienced interns. Consequently, patients may not take us seriously, and their responses to our inquiries may lack patience and accuracy. For instance, during a recent assessment, I identified the need to prepare a patient for surgery, including advising them to be fasted (NPO) [author:* “NPO” stands for “nil per os,” which is a Latin phrase meaning “nothing by mouth.” In medical terminology, it is used to indicate that a patient should abstain from eating or drinking for a specified period, typically before a medical procedure or surgery*] as part of the nursing process. However, when I communicated this information to the patient, they didn't regard it seriously, and later, I discovered that they had even consumed some water.” (Participant Two*).

Or another student said in this regard: *“I encountered a situation where I had the responsibility to change a patient's dressing to prevent infection. However, the ward staff redirected me to the ultrasound ward to assist with another patient who was not under my care. I felt unable to refuse this task, as I did not perceive myself as having the necessary independence (Participant Five)."*

Another student expressed their lack of authority and autonomy to carry out various nursing interventions: *“At the hospital, when we attempt to contact the doctor for a consultation regarding a patient's condition based on nursing diagnosis, we are often not permitted to do so and are told to wait until the doctor arrives themselves, as recounted by Participant Seven."*

Hence, it appears that nursing students grapple with a blurred distinction between their roles as learners and aspiring professional nurses during their internship. They often face challenges in performing various nursing interventions autonomously. Perhaps it is time to reconsider the internship student's role description, permitting them to execute all patient-related interventions under experienced nurse supervision. This adjustment could foster self-confidence, enhance learning, and better prepare students for a seamless transition into the nursing profession.

#### 3.1.2 Insufficient proficiency in the nursing process

Another factor impeding students' utilization of the nursing process in patient care is their inadequate knowledge and experience in applying it, which appears to be linked to their prior years of education. Students have reported encountering difficulties in identifying nursing diagnoses or determining appropriate interventions for specific nursing diagnoses. This problem can be attributed to inadequate and erroneous instruction in the nursing process over the past three years by some educators. Additionally, some students may not have invested sufficient effort in learning during previous semesters. These factors collectively contribute to students lacking the necessary knowledge and skills for proficiently analyzing and applying the nursing process. As one professor noted: *“Some of our colleagues do not prioritize the nursing process and fail to instruct students accordingly, resulting in students lacking the necessary skills to effectively implement the nursing process when they commence their internship, as noted by Supervising Professor 2."*

A student also said: *“I had the nursing process lesson in the first semester, but in the subsequent semesters, when we attended clinical rotations, the professors didn't require us to apply the nursing process in our patient care. This resulted in me having a poor understanding of the nursing process, not valuing its importance, and encountering difficulties in its application.” (Participant 11*).

Another student said: *“I personally struggled with recognizing a patient's issue and translating it into a nursing diagnosis. For instance, I would observe symptoms like infection or bleeding, but I lacked the knowledge of how to formulate a nursing diagnosis.” (Participant 3*).

Hence, it becomes apparent that certain challenges surrounding the utilization of the nursing process during the internship can be traced back to earlier stages of university education. It's essential to acknowledge that if we aim to produce well-educated and highly proficient nursing students who consistently apply the nursing process in their care practices, it is imperative to ensure that the quality and principles of our professors' education are of the highest standard. Consequently, there may be a need for a more thorough examination and heightened emphasis on the teaching of the nursing process throughout the educational journey.

#### 3.1.3 Motivational deficiency

Another significant factor contributing to the underutilization of the nursing process is the lack of motivation among students in the field of nursing. Multiple factors lead to conflicting and lofty expectations from others, uncertainty about their roles, and at times, discouragement from colleagues in the nursing staff. These issues can result in reduced motivation and a sense of ambiguity about their future in the field of nursing. Consequently, some students who are contemplating leaving the nursing profession may not take the nursing process seriously, perceiving it as futile and superfluous work.

A student said: *“When I began my internship and witnessed the hospital nursing environment, I was taken aback. It appeared that our tasks were not exclusive to nurses, as many aspects of patient care involved the patient's family. This experience led to a sense of disillusionment with nursing for me.” (Participant 5*).

Another student, concerning the issue of insufficient motivation, expressed the following viewpoint: *"My primary hindrance in effectively executing the nursing process was a severe lack of motivation.* Nurses *around me questioned the choice of this profession, claiming it went unnoticed and had no prospects. These discouraging remarks made me question the purpose of putting in so much effort to implement the nursing process. I contemplated discontinuing my nursing education, so I would simply complete my shifts and focus on obtaining grades.” (Participant 1).*

An essential factor contributing to nursing students' incomplete or non-use of the nursing process is the observation of experienced nurses in hospital settings. At times, nursing students witness established nurses who either do not employ the nursing process or hold reservations about its efficacy. Instead of motivating and supporting students in their efforts to provide care guided by the nursing process, some nurses may mock them or suggest skipping certain process steps or resorting to copying from previous records to save time. It's worth noting that this behavior can stem from various reasons, including the high patient-to-nurse ratio in Iranian healthcare, inadequate resources, insufficient support from superiors, among others. Further investigation is warranted to delve into these underlying causes.

In this context, one of the students said: *“Some of the nurses themselves appear to have limited familiarity with the nursing process. Additionally, I've noticed a lack of thorough evaluation in their care practices. For instance, they administer painkillers but fail to assess whether the patient's pain has actually improved. This could be due to their heavy workload and time constraints. When I observe these practices, I find myself questioning the significance of the nursing process. It seems possible to provide extensive care without adhering to the process meticulously, and it's unclear whether anyone would even recognize whether I've implemented the nursing process or not.” (Participant 5*).

Respected nurses in teaching hospitals and nursing professors, who interact with students, should recognize the profound impact of their actions and behaviors on students' motivation. While the nursing profession may face its share of challenges, demotivating students or encouraging them to abandon their pursuits is not the solution. Instead, focusing on the more effective and precise education of nursing students can pave the way for the advancement and growth of the nursing profession in the future. This approach is essential for nurturing a skilled and dedicated nursing workforce.

### 3.2 Organizational challenges

Another factor contributing to the underutilization of the nursing process during nursing students' internship is associated with the hospital environment and its nursing staff. These factors can be categorized as organizational factors and are outlined below:

#### 3.2.1 Shortage of nursing staff

One of the most pressing challenges in Iran's public hospitals is the overwhelming number of patients admitted to the wards, coupled with an insufficient nursing staff. Consequently, the nurse-to-patient ratio in Iran falls significantly below international standards. This situation leads to each nurse being responsible for a larger number of patients during each shift, making it virtually impossible for nurses to fully and comprehensively execute all phases of the nursing process. Nurses are compelled to provide only quick assessments, focus on basic and limited diagnoses, attend to essential patient care tasks during the implementation phase, and, in many instances, find it extremely challenging to carry out the evaluation phase.

This predicament holds true even for nursing students during their clinical placements, as they are often assigned a substantial caseload of patients, leaving them unable to complete all the steps of the nursing process thoroughly and accurately. As one student pointed out: *“When nurses have a smaller patient load, they can readily provide comprehensive care, including patient education, massages, repositioning, and more. However, with the current situation of a high patient-to-nurse ratio due to a shortage of nursing staff, some responsibilities, like tube feeding, changing dressings, and even suctioning patients, are often delegated to the patient's family. Nurses, in turn, are left with limited time, primarily for medication administration and documentation in patient files. Consequently, the complete nursing process is not effectively implemented.” (Participant 4).*

Another student mentioned*: “I encountered a situation where a patient was receiving a heparin drip, and the nurse had documented that education about the risk of bleeding was provided to the patient. However, the patient had not received any such information. The nurse simply wrote it down without actually having the time to educate the patient. This scenario has occurred multiple times during my experiences, where I've recorded interventions that I haven't genuinely carried out. It leaves me feeling a sense of guilt, but the sheer volume of work leaves me with no alternative.” (Participant 1*).

It is undeniable that the presence of an adequate number of nursing staff in a ward, ideally approaching international standards, significantly enhances the quality of nursing care. With ample time, nurses can deliver comprehensive and thorough care by meticulously executing all phases of the nursing process. However, when time is limited due to understaffing, nurses may only be able to address routine and basic care tasks, leaving more complex aspects of patient care unattended.

#### 3.2.2 Suboptimal interpersonal dynamics

It's well-known that the nursing process comprises five stages that often require collaboration and participation from various stakeholders. Some nursing interventions are inherently interdisciplinary in nature. However, during interviews with both students and professors, it has become evident that interdisciplinary cooperation within the field of nursing in Iran is often insufficient and suboptimal. The absence of effective collaboration among nurses themselves, with doctors, patients, and their families results in students, just like their colleagues working as ward nurses, settling for the bare minimum in patient care. They struggle to provide more comprehensive and professional care.

Furthermore, this lack of interdisciplinary interaction dampens students' motivation. They begin to feel that nursing work is undervalued or overlooked by others, which subsequently leads to them being less attentive and less meticulous in their practice. Below are some examples from participants' statements illustrating these concerns: *“At times, the nursing staff don't perceive us as colleagues but rather as individuals they can rely on to handle their tasks. They often pressured us to perform their duties, such as taking complete ECGs and vital signs for the patients. These additional responsibilities consumed a significant amount of time and made it challenging for me to execute the nursing process thoroughly.” (Participant 4*).

Another student said*: “Nurses with extensive experience often dismiss the nursing process entirely. They neither utilize it themselves nor accept our attempts to implement it. In some instances, they pressurize us to perform tasks that seem futile, and if we resist, they report our actions to the professors. Interestingly, even our professors may* not *be acknowledged by these seasoned nurses, who claim that teaching differs significantly from real-world hospital situations. Consequently, we find ourselves caught between the perspectives of the nurses and our professors.” (Participant 6).*

Regrettably, the gap between education and clinical practice persists in some hospitals and healthcare facilities, with clinical staff sometimes isolating themselves from the academic and university environment. In certain cases, the contributions of professors and educational staff are undervalued. It is imperative to find solutions that enhance communication and collaboration between universities and hospitals, as well as among various healthcare professions, including medical doctors, physiotherapists, pharmacists, and nurses. Fostering greater interdisciplinary teamwork and communication has the potential to significantly improve the overall quality of patient care. This can be achieved through initiatives that promote mutual understanding, shared knowledge, and a cohesive approach to healthcare delivery.

### 3.3 Gradual erosion of the nursing process

As observed, nursing students often begin their hospital experiences with a strong commitment to implementing the nursing process thoroughly. However, over time, their dedication tends to diminish, primarily due to factors that appear to be predominantly associated with management and supervisory shortcomings. These factors encompass an overemphasis on the theoretical aspects of the nursing process, inadequate student supervision, inconsistencies in teaching approaches among professors, and a predominant focus on nursing diagnoses and interventions by the nursing staff, while neglecting the holistic clinical application of the process.

Consequently, students gradually lose their sensitivity and commitment to implementing and applying the nursing process, resulting in a reduced level of seriousness and engagement with this essential aspect of their practice. Addressing these managerial and supervisory issues and fostering a more comprehensive and practical approach to the nursing process could help reignite students' dedication to its effective use in patient care.

A student said: “*I've observed* numerous *shift changes, and it's clear that the nursing process often receives minimal attention. The supervisor primarily checks superficial aspects like the cleanliness of the patient's bed, the condition of the IV line, and the status of the IV drip. Consequently, when I witness that managers and supervisors prioritize these aspects over patient education and care, it leads me to place more emphasis on the physical appearance of the patient's bed and bedding than on their overall well-being and healthcare needs.” (Participant 6).*

Another student said: “*Most of the ward's cardexes only have a section for nursing diagnosis, with no sections dedicated to patient assessment, intervention planning, implementation of nursing care, or evaluation. As a result, both hospital staff and myself as a student tend to focus primarily on documenting nursing diagnoses, while the other critical aspects of the nursing process receive less attention.” (Participant 1*).

Another student highlighted an important observation regarding the routinization of the nursing process and noted: *“I've witnessed on numerous occasions that nurses tend to apply the same nursing diagnoses for all patients, almost as if some diagnoses have become standard routines. For instance, pressure ulcers or the risk of falling from the bed are frequently assigned. Whether by choice or out of necessity, when we find ourselves in such a work environment, our approach to the nursing process gradually shifts away from the thoroughness and dedication seen in the initial days, and we begin to rely more on a series of routine and customary actions.” (Participant 7).*

It is evident that despite significant advancements in promoting the application of the nursing process in hospital and clinical settings, there is still room for improvement. Educational managers and hospital supervisors, in collaboration with university professors, should proactively devise innovative solutions to prevent the nursing process from becoming a routine and mundane task. These solutions may include revamping nursing cardexes, organizing educational workshops focused on the nursing process, and implementing more comprehensive and vigilant oversight of the critical facets of the nursing process. By fostering a culture of continuous improvement and emphasizing the value of the nursing process, healthcare institutions can ensure that nursing care remains patient-centered, evidence-based, and highly effective.

## 4 Discussion

The objective of this study was to examine the barriers and difficulties encountered in the application of the nursing process, as perceived by nursing internship students and their supervising professors.

One significant challenge identified in implementing the nursing process, based on the experiences of the internship students, was their lack of practical independence. Students often found themselves still perceived as novices rather than fully-fledged colleagues, which resulted in limited opportunities to engage in various nursing interventions and follow-up activities. This lack of trust from both patients and colleagues undermined students' self-confidence, motivation, and enthusiasm, ultimately hampering their ability to effectively implement the nursing process. This sentiment aligns with a study by Günay et al., where students expressed a strong desire to be recognized as competent nurses rather than mere trainees, highlighting the importance of addressing issues related to practical independence and professional identity during nursing education and clinical training [42]. The study by Kalyani et al. underscores that the negative attitudes of nurses towards the presence of students in the ward, coupled with the lack of support and trust from both nurses and patients, collectively contribute to the creation of a non-supportive atmosphere for nursing students. This challenging environment can be detrimental to students' learning experiences and their ability to effectively apply the nursing process during their clinical training. Addressing these issues and fostering a more supportive and collaborative atmosphere in healthcare settings is essential for enhancing the education and development of nursing students [43].

The lack of sufficient knowledge in applying the nursing process emerged as a prominent issue from participants' experiences. Students encountered difficulties in formulating nursing diagnoses, often resorting to repeating predetermined diagnoses when communicating with their supervising professors. This knowledge gap was attributed to ineffective teaching methods in theoretical classes, professors' insufficient emphasis on the nursing process during past semesters in clinical settings, and a lack of proper supervision. These factors contributed to a gradual desensitization to the nursing process. Similarly, in Jasemi et al.'s study (2013), students voiced their struggle with the disparity between intensive theoretical training received during internships and the challenges of translating this knowledge into practical skills. The disconnect between theoretical instruction and real-world hospital practices was a recurrent issue, creating a gap between classroom learning and clinical experiences [44]. Agyeman-Yeboah et al. (2017) identified one of the reasons for this gap as the limited knowledge of nursing professors about the nursing process itself. This lack of understanding hinders their ability to effectively teach the nursing process, contributing to the divide between theoretical education and practical application [45]. According to a study conducted in Iran, a significant majority (75.6 %) of instructors used routine teaching methods to instruct the nursing process. This suggests a prevalence of conventional and potentially less effective teaching approaches in nursing education in Iran [46]. Incorrect training methods can indeed result in insufficient knowledge among nursing students in the field of the nursing process. When teaching strategies do not effectively convey the principles and practical applications of the nursing process, students may struggle to grasp the concepts and procedures required for competent nursing care. It underscores the importance of employing pedagogically sound and clinically relevant teaching methods to equip nursing students with the necessary knowledge and skills [47]. Indeed, having proper knowledge about the concept and how to implement the nursing process is crucial for its effective implementation. Without a solid foundation of knowledge, nursing students may struggle to understand the underlying principles, make accurate nursing diagnoses, plan appropriate interventions, and evaluate patient care. Therefore, prioritizing comprehensive and accurate education on the nursing process is essential to ensure that students can apply it competently in clinical practice [48].

Students' diminished interest in nursing and their low motivation posed additional challenges in implementing the nursing process. Many students expressed intentions to pursue alternative career paths for financial reasons after graduation. In clinical learning, individual motivation serves as a vital internal factor that can significantly impact the learning and application of nursing skills [49]. Several reasons have been identified for the decline in motivation among nursing students in various studies. One of these factors is educational injustice and discrimination experienced by nursing students compared to their medical counterparts in clinical learning environments. For example, in studies by Kalyani et al. (2019) and Hadian Jazi (2022), students reported instances where nurses treated them unfairly, such as taking away a patient file from a nursing student but not from a medical student. Additionally, nursing students were occasionally assigned tasks typically associated with non-medical roles, like cleaning tables, which contributed to feelings of inequity and reduced motivation [43,50]. These external factors can significantly impact students' enthusiasm for pursuing a career in nursing [50,51].

According to the perspective of internship students, a key challenge in implementing the nursing process in Iran is the insufficient facilities and the imbalance between the number of nurses and patients. This issue was highlighted in Fernández-Sola et al.'s study (2011), where participants reported a lack of resources, references, and access to computers in their departments, as well as limited financial resources for teaching the nursing process. This situation is especially challenging in developing countries where resource constraints further exacerbate the difficulties in nursing education [52]. In the study by Zamanzadeh et al. (2015), it was noted that the shortage of time resulting from the high patient load can hinder the proper implementation of the nursing process. This underscores the challenge of providing comprehensive nursing care when healthcare providers are overwhelmed with a large number of patients [48].

This study also highlighted the issue of role modeling by nurses who lacked sufficient knowledge about the nursing process. Rezakhani et al.'s study (2020) emphasized that nurses with insufficient knowledge and non-scientific care practices are not suitable role models for students. Moreover, ward nurses often face time constraints and may not be able to provide practical guidance to students due to their heavy workload and potentially outdated information. This lack of effective role modeling can hinder students in their learning and implementation of the nursing process [53]. Therefore, it is highly recommended that nurses take proactive steps to update their knowledge in the field of the nursing process, both in theoretical and practical aspects. This continuous learning and enhancement of skills will not only benefit their own practice but also serve as better role models for nursing students, ultimately improving the quality of nursing education and patient care [54].

The lack of effective interactive dynamics posed another challenge in the implementation of the nursing process. According to students, communication among nurses for the proper execution of the nursing process is severely limited. Furthermore, their interactions with students in this regard are often inadequate, treating students more as additional labor to handle various ward tasks and nursing responsibilities. This results in a waste of students' time and presents a significant challenge in their efforts to implement the nursing process. Given that internship students are still relatively new to the clinical environment and may lack the efficiency and speed of experienced nurses, being assigned additional tasks beyond their core responsibilities consumes a significant amount of their time. This added workload can significantly impede their ability to implement the nursing process effectively for their patients.

## 5 Implications for research/practice

The nursing process is a systematic and highly precise scientific approach that enables comprehensive patient assessment, accurate diagnosis, appropriate intervention implementation, and subsequent evaluation to achieve desired patient care outcomes. Implementing this process consistently in nursing care has the potential to save time, resolve patient issues, reduce hospitalization duration, and decrease healthcare costs for both patients and governments.

It is therefore recommended that further extensive research be conducted on the implementation of the nursing process among nurses and nursing students, who are soon to enter clinical work environments. The findings from such studies can inform curriculum planning for nursing process education in universities. Identifying and addressing similar challenges in nursing process implementation among students can help advance the professionalization of nursing and improve patient care standards.

## 6 Limitations of study

This study has certain limitations, particularly regarding the generalizability of its findings to broader populations and different student groups in various locations. This limitation is inherent in qualitative research. In qualitative inquiry, the emphasis is often on depth rather than breadth, and findings are typically context-specific rather than generalizable to a larger population [55].

The small sample size in qualitative research is intentional and allows for an in-depth exploration of the experiences, perspectives, and meanings of the participants within a specific context [56]. While the findings may not be applicable to a broader population, they offer valuable insights and contribute to theoretical development and understanding within the specific context under study. Therefore, it is crucial to interpret the findings of qualitative research within the context in which they were generated and to recognize the limitations regarding generalizability. Future research may benefit from replicating the study with larger and more diverse samples to explore the transferability of findings to other contexts.

Nevertheless, the insights gleaned from this study can offer valuable perspectives for educators, curriculum planners, and teaching hospital administrators. These insights can guide efforts to address similar challenges in nursing education and clinical practice, even if they cannot be universally applied.

## 7 Conclusion

The significance of implementing the nursing process in the nursing profession, to provide higher quality patient care, is widely acknowledged. Its use by nurses not only saves time in patient care but also effectively addresses critical patient issues, ultimately reducing hospitalization costs and enhancing nurse job satisfaction. Therefore, it is crucial to identify challenges in implementing the nursing process, particularly among nursing students who are preparing to enter clinical settings.

This study has revealed that such challenges often stem from the dual roles of nursing students, as they navigate between being a student and an aspiring nurse, sometimes not being taken seriously by others. Additionally, challenges can arise from issues within hospitals, specific wards, and personnel, which can erode the motivation and resilience of nursing students in clinical settings. There may also be environments where the nursing process is neglected, overlooked, or actively discouraged by nursing staff, clinical managers, and even medical professionals.

As such, it is imperative for educational institutions, hospitals, and curriculum planners to remain cognizant of these challenges and actively work towards their resolution. By doing so, they can promote the extensive utilization of the nursing process, benefiting everyone involved, from patients to nurses, and even government healthcare systems.

## Ethics statement

This research was proposed at the Study Center of Isfahan University's Nursing Faculty and received approval from the ethics committee with code IR. MUI.NUREMA.REC.1401.125.

In this study, both oral and written consent were obtained from each participant after explaining the study's purpose. All participants were assured of the complete confidentiality of their interviews, and recordings of the interviews were scheduled for destruction after transcription and analysis. Additionally, nursing students were informed that their comments regarding the use or non-use of the nursing process would not impact their grades. It is confirmed that informed consent was obtained from the participants for the publication of all data and other data included in the manuscript. And it is confirmed that the study complies with all regulations.

## Funding

This study was financed by the Vice Chancellor for Research of 10.13039/501100003970Isfahan University of Medical Sciences (Project number 2401147).

## CRediT authorship contribution statement

**Amir Shahzeydi:** Writing – original draft, Methodology, Investigation. **Zahra Hadian Jazi:** Writing – review & editing, Methodology, Formal analysis, Conceptualization. **Sima Babaei:** Supervision.

## Declaration of competing interest

The authors declare that they have no known competing financial interests or personal relationships that could have appeared to influence the work reported in this paper.
