# Supplementary material for: Exploring the perceptions of nursing internship students and their faculty mentors regarding the complexities and hindrances associated with implementing the nursing process within the clinical setting: An in-depth qualitative investigation
Source: Heliyon. 2024 May 29;10(11):e31715. doi: 10.1016/j.heliyon.2024.e31715 (PMC11168315; doi:10.1016/j.heliyon.2024.e31715)
Supplement: Multimedia component 1 [file mmc1.docx]

**Please keep in mind that all the interviews were conducted in Farsi. And the participants' questions and answers were informal and conversational, which became more formal after being translated into English.**

**Also, in some statements, Iranian culture and the atmosphere of Iranian hospitals have made the interviewer understand the depth and meaning of the participant, which may be a little difficult for you, dear referee, considering the lack of familiarity with Iranian culture.**

*******

**Question:** **Describe your initial encounter with the nursing process. When did you first become familiar with it? Please elaborate.**

**Participant’s answer:** My introduction to the nursing process occurred during my second semester. Initially, I viewed it from a theoretical perspective, assuming it would be a non-issue after the exam. However, when practical training in a hospital ensued, professors requested that we engage in patient care, emphasizing the importance of nursing diagnoses and evaluations. This shifted my perception, highlighting the practical nature of the nursing process. Yet, during my internship, I observed hospital staff neglecting proper adherence to the nursing process. Attempts to consult doctors for new nursing care were often met with resistance, being told to wait for the doctor.

**In your perspective, what are the pros and cons of employing the nursing process? Specifically, what advantages and disadvantages have you encountered in your own experiences over these past few years? Allow me to elaborate.**

On the positive side, the nursing process alleviates confusion. When I assess and examine a patient during the first shift, it provides clarity regarding the patient's issues (nursing diagnosis) and outlines the tasks I, as a nurse, need to undertake (nursing implementation).

Furthermore, the nursing process aids in prioritizing the patient's problems. For instance, if a patient is in severe pain, the process ensures that we address crucial issues first, avoiding the oversight of more detailed matters like examining pressure ulcers.

Another advantage lies in the increased time nurses spend in the ward with the patient. When different shifts are assigned the same patients, the continuity allows for better follow-up and enhanced care. I've experienced this firsthand as a nurse, working within the framework of the nursing process. It enabled me to track my patient's problems, identify solutions, and determine where to focus my attention—resulting in highly effective care.

The nursing process also serves to minimize errors, be it in medications or treatments. Failure to adhere to the process can lead to oversights, such as neglecting the initial stage of patient examination, which can result in medication errors or falls. Strict adherence to the nursing process is essential to avoid such mistakes and ensure comprehensive care.

Additionally, if nurses adhere to standardized processes, their work becomes more professional. While some may perceive nurses as individuals solely responsible for administering injections and medication, adherence to the nursing process showcases the profession's depth and professionalism beyond these tasks.

Patients also tend to communicate more effectively when the nursing process is followed. By uncovering the patient's problem through this systematic approach, care becomes specialized rather than routine. This not only prevents oversights in patient care but also contributes to the professionalization of the nursing field, emphasizing the nurse's autonomy in decision-making and the application of scientific principles, moving beyond mere adherence to doctor's instructions.

**Question: Can you discuss specific obstacles or difficulties you have faced when utilizing each stage of the nursing process (assessment, diagnosis, planning, implementation, evaluation) in clinical practice?**

One of the significant challenges we encountered was the resistance from fellow nurses who either didn't adhere to the nursing process or made light of our attempts to implement it accurately. Despite this, I chose to stay true to myself and not be deterred by external opinions. Many nurses were unfamiliar with the nursing process, and there was a lack of patient evaluation. For instance, administering painkillers without subsequent pain assessments was common, possibly due to their busy schedules.

Patient cooperation was another hurdle, as many patients grew impatient with repeated questions from doctors and nurses. Additionally, we often faced the challenge of a high patient load, making it difficult to thoroughly examine and provide proper care according to the nursing process.

Another drawback was the timing of the evaluation stage, which couldn't always be completed within the same shift. Conditions requiring follow-up, such as electrolyte imbalances, demanded more time. Unfortunately, due to forgetfulness or patient changes, these evaluations were sometimes neglected.

The internship, intended as an opportunity to apply classroom learning at the bedside, presented challenges as I looked up to the ward nurses as role models. Discrepancies in practices, such as the use of sterile dressings, arose when observed at the bedside. Time constraints and inadequate facilities often influenced the adoption of these practices.

Despite attempts to incorporate the nursing process at the bedside, the realities differed from what was taught in textbooks. Obstacles included the sheer number of patients, time limitations, and a decline in motivation, with many nurses seeking to complete shifts quickly and hand over patients promptly. The frequent rotation of departments on a monthly basis added another layer of difficulty.

Moreover, supervisors in the hospital had distinct expectations compared to those in the university. The hospital's workload and time constraints often dictated different procedures, deviating from what was outlined in academic materials. Their rationale was that the workload was excessively high, justifying deviations from textbook practices

**Question: Considering the challenges faced during your internship, what factors could facilitate the implementation of the nursing process?**

Based on my own experiences during this period and the challenges faced, here are factors that can facilitate the implementation of the nursing process in internships:

1. **Repetition and Reinforcement:** Repetition plays a crucial role in solidifying knowledge. Regularly revisiting the nursing process, along with a focus on specific aspects like pressure sore care, helps embed essential practices in the minds of nurses. Frequent repetition fosters familiarity and mastery.
2. **Emphasis on Importance:** Understanding the significance of the nursing process is paramount. Education and awareness campaigns should underscore its importance in ensuring systematic and scientific patient care. This can contribute to a collective acknowledgment of the value it brings to the nursing profession.
3. **Scientific Training:** Formal and structured scientific training programs dedicated to the nursing process can enhance comprehension and application. By incorporating practical scenarios and case studies, nurses can better grasp the real-world implications and benefits of adhering to the nursing process.
4. **Recognition and Incentives:** Recognition and encouragement play a vital role in motivating nurses to excel in implementing the nursing process. Creating a system where individuals who perform exceptionally well are acknowledged, perhaps through incentives or points, can instill a sense of accomplishment and pride.
5. **Strategic Patient Assignment:** Schedulers should carefully distribute patients during shifts to ensure a manageable workload. A logical patient assignment strategy prevents nurses from being overwhelmed, allowing them sufficient time to apply the nursing process thoroughly and effectively.
6. **Increase in Staff Numbers:** Increasing the number of nurses can alleviate the workload burden. With a higher nurse-to-patient ratio, nurses can dedicate more time to each patient, enhancing the quality of nursing care. This approach promotes a focus on individualized patient needs and comprehensive care.

By implementing these measures, there is potential to create an environment that supports the effective application of the nursing process during internships, fostering a culture of excellence and scientific rigor in nursing care.
